# Models of Governance of Disability Therapy Support Workers in Rural and Remote Settings: A Systematic Scoping Review

**DOI:** 10.3390/ijerph21060693

**Published:** 2024-05-28

**Authors:** Anna Moran, Kim Bulkeley, Genevieve Johnsson, Elaine Tam, Catherine Maloney

**Affiliations:** 1 Department of Rural Health, Melbourne Medical School, The University of Melbourne, Shepparton, VIC 3630, Australia; 2 Faculty of Medicine and Health, School of Health Sciences, The University of Sydney, Camperdown, NSW 2006, Australia; kim.bulkeley@sydney.edu.au (K.B.); genevieve.johnsson@sydney.edu.au (G.J.); elaine.tam@sydney.edu.au (E.T.); 3 Services for Australian Rural and Remote Allied Health, Barton, ACT 2600, Australia; catherine@sarrah.org.au

**Keywords:** disability, support worker, assistant, therapy, governance, rural, remote, realist synthesis, allied health, remote, risk, quality

## Abstract

The National Disability Insurance Scheme (NDIS) ushered in a transformative era in disability services in Australia, requiring new workforce models to meet evolving participant needs. Therapy Assistants are utilised to increase the capacity of therapy services in areas of workforce shortage. The governance arrangements required to support this emergent workforce have received limited attention in the literature. This review examined the key components and contextual factors of governance in rural settings, specifically focusing on therapy support workers under the guidance of allied health professionals in rural and remote areas. Guided by the social model of disability and the International Classification of Functioning, Disability and Health, a realist perspective was used to analyse 26 papers (after deduplication), mostly Australian and qualitative, with an emphasis on staff capabilities, training, and credentialling. Success measures were often vaguely defined, with most papers focusing on staff improvement and few focusing on client or organisational improvement. Consistent staffing, role clarity, community collaboration, and supportive leadership were identified as enabling contexts for successful governance of disability therapy support workers in rural areas. Investment in capability (soft skills) development, tailored training, competency assessment, credentialling, and supervision were identified as key activities that, when coupled with the identified enabling contexts, were likely to influence staff, client and organisational outcomes. Further research is warranted to explore long-term impacts of governance arrangements, educational program accountability, and activities targeted at enhancing staff capabilities.

## 1. Introduction

Workforce development in the National Disability Insurance Scheme (NDIS) is a changing environment, with new models of service delivery responding to participant needs. The Therapy Assistant model is one such emerging model.

The National Disability Insurance Scheme is a major social policy reform impacting the lives of people with a disability in Australia, providing supports to increase participation and inclusion in the community. The NDIS Integrated Market Sector and Workforce Strategy [1] has highlighted the need for significant expansion of a skilled and adequately distributed workforce to enact the aspirations of the NDIS, with a particular mention of projected shortages of allied health workers as the scheme rolls out. However, 58% of organizations had plans to expand the therapeutic support segments of their business, and over 73% of disability service providers report difficulties recruiting (and retaining) allied health professionals [2].

The allied health assistant model has been supported in policy and funding frameworks in the NDIS, with an emerging uptake by disability service providers in response to these two drivers [3]. However, there has been scant attention paid to the adaptation of service delivery models and governance frameworks to support this emerging model of practice. There are many lessons to be learned from existing health based allied health assistant frameworks that address risk, quality, supervision, competency assessment, training, and scope of practice [4,5]. However, the social model of disability and a focus on participation and inclusion requires a more nuanced and contextualized framework to support allied health assistant models in the disability sector.

The quality and sustainability of the disability workforce is a crucial component of the success of the NDIS. The NDIS has enhanced the quality of life of many participants; nonetheless, a number of stakeholders continue to express concerns that several factors are impacting the quality of services provided under the NDIS. These include changes in workplace culture, a high turnover of staff, the increasing casualization of the workforce due to market pressures, a lack of professional development and training, and regional and rural workforce shortages [6]. These issues are compounded by the NDIS pricing regime, which has made it financially harder for service providers to address these quality issues and support complex clients [6].

In the NDIS, therapy supports are for participants with an established disability, where maximum medical improvement has been reached, to facilitate functional improvement. For people who access the Scheme as ‘early intervention’ NDIS participants, reasonable and necessary supports are likely to be a blend of medical and disability therapies but should be predominantly disability therapy supports. Therapy in this context must be aimed at adjustment, adaption, and building capacity for community participation.

The International Classification of Functioning (ICF) provides a framework that helps to shift the focus of the allied health assistant role from a medical model focused on impairments and health conditions to a social lens focused on inclusion and participation [7]. Evidence demonstrates that allied health assistants, and, more broadly, support workers, with the right balance of social, emotional, and technical skills are ideally placed to support a social inclusion model of care [8].

This change in focus necessitates an equal shift in focus around how service delivery models are devised and delivered and the governance frameworks that are applied to allied health assistants in the disability sector. The scope of practice, approaches to supervision, assessment of competency and training, and the collaboration between allied health assistants and therapists require rethinking in long-term disability settings with supports provided in a diverse range of natural contexts. The inclusion of remote practice options such as telepractice and intermittent contact between allied health professionals and allied health assistants require additional consideration to ensure the maintenance of quality services, to mitigate risk, and to optimize conditions where client needs are met.

The objective of this study is to therefore identify different governance models for disability therapy support workers in rural and remote settings; to better understand the contexts and mechanisms that underpin these models; to better understand what success looks like; and to better understand how these elements come together and interact to influence their “success”.

Given the complexity of factors that can influence the development, implementation, and outcomes of governance frameworks, this study is underpinned by a logic framework [9,10] to identify and illustrate how different elements of governance frameworks and associations between elements may impact on the outcomes and ultimately the ‘success’ of a governance framework for disability therapy support workers.

This scoping review is part of an ongoing program of research that aims to produce a robust, evidence- and consumer-informed disability therapy and support worker governance framework for rural and remote disability workers who deliver allied health interventions.

A preliminary search of PROSPERO, the Cochrane Database of Systematic Reviews, and JBI Evidence Synthesis was conducted, and no current or underway systematic reviews on the topic were identified.

## 2. Review Questions

Using a realist perspective [10,11], the overarching review questions are as follows: What models of governance exist for rural and remote disability support/therapy workers who deliver allied health interventions? And what models work best, for whom, in which circumstance, and why?

In order to answer this question, the following sub-questions were identified:

- What governance models for disability support/therapy workers in rural and remote settings exist and what are the key components of these models?
- What contexts and mechanisms are required to successfully apply governance models for disability therapy support workers (DTSW) in rural and remote settings?

## 3. Materials and Methods

A scoping review methodology was applied in accordance with the Joanna Briggs Institute guidance for undertaking scoping reviews [12].

### 3.1. Inclusion Criteria

#### 3.1.1. Participants

The review considered all governance models pertaining to allied health assistants, therapy assistants, and DTSW who deliver support to persons with disabilities under the guidance of allied health professionals. An allied health profession was defined as one that had the following [13,14]:

- A direct patient care role and may have application to broader public health outcomes;
- A national professional organization with a code of ethics/conduct and clearly defined membership requirements;
- University health sciences courses (not medical, dental, or nursing) at Australian Qualifications Framework (AQF) Level 7 or higher, accredited by their relevant national accreditation body;
- Clearly articulated national entry level competency standards and assessment procedures;
- A defined core scope of practice;
- Robust and enforceable regulatory mechanisms;

and who met the following criteria:

- Are autonomous practitioners;
- Practice in an evidence-based paradigm, using an internationally recognized body of knowledge to protect, restore, and maintain optimal physical, sensory, psychological, cognitive, social, and cultural function;
- May utilize or supervise assistants, technicians, and support workers.

This definition was used to identify allied health professions that are used predominantly in the disability sector to deliver therapy interventions (see Additional File S1 for included allied health professions and source of evidence for their role in the disability sector). These professions included Allied health worker, Allied Health professional, Physiotherapist, Occupational therapist, Speech pathologist, Psychologist, Dietitian, Exercise physiologist, Speech pathologist, Podiatrist, Social worker, Therapist, Skilled therapist, and Aboriginal and Torres Strait Islander Health worker.

Support workers have traditionally worked to fill gaps in service provision; therefore, their roles are often designed to be as flexible as possible. As such, the range of terms used to describe support workers are as diverse as the roles they perform [9]. Role flexibility is even more apparent in the disability sector and in rural settings where generalist roles are commonplace [15,16,17,18,19]. Support workers working in delivering disability therapy support in rural settings are therefore more likely to be performing multiple and varied roles across multiple settings with multiple different stakeholders. As such, the terminology for support workers who deliver therapy interventions aligned with allied health in the disability setting continues to evolve. To capture the diversity of the roles of the disability therapy support workforce, Additional File S1 describes the range of titles that are currently used with a corresponding reference source. These titles were used to inform the range of search terms used for this review.

#### 3.1.2. Concept

**Figure 1:**
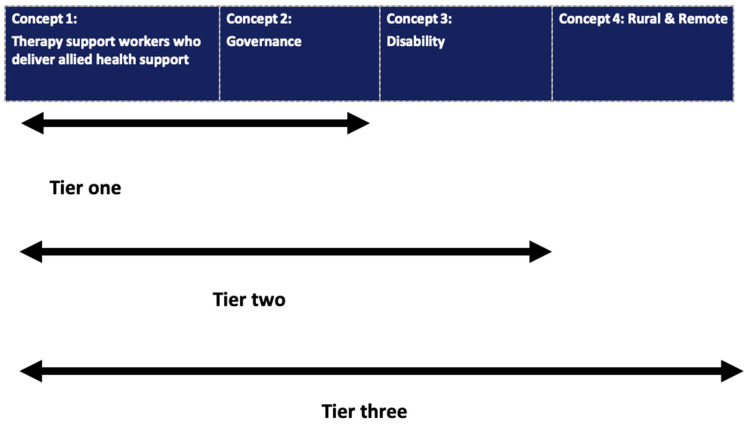
Concepts and evidence sampling tiers.

The review considered four key concepts (Figure 1). Concept one is therapy support workers who deliver allied health supports; concept two is governance interventions and governance models relating to therapy support workers who deliver support under the guidance of allied health professionals; concept three is the disability setting; concept four is the rural and remote setting. Combining these four concepts, the review considered studies that explored evidence relating to governance and governance models of disability therapy support workers who deliver support under the guidance of allied health professionals in rural and remote settings. The review is theoretically underpinned by the social model of disability [20], focusing on the governance of care and interventions that reflect the ICF core areas of participation and inclusion [7].

Clinical governance encompasses activities aimed at improving the quality and safety of care. Scally and Donaldson [21] define clinical governance as “a system through which organizations are accountable for continually improving the quality of their services and safeguarding high standards of care by creating an environment in which excellence in clinical care will flourish”. A clinical governance framework is therefore a set of initiatives designed to enhance quality and the promotion of a productive culture and climate within which good outcomes can be achieved [9]. Braithwaite and Travaglia [9] identify that successful governance frameworks require key values, organizing and governing structures, organization and use of data and evidence, and sponsoring a person-centred focus as central concepts for effective governance.

In Australia, in the disability context, these governance principles are reflected in the NDIS (Quality Indicators) Guidelines 2018 [22] and NDIS code of conduct [23]. These are overseen by the NDIS quality and safeguards commission. A number of terms that are used to describe elements of governance that are specific to the support workforce and general terms for governance are listed in Additional File S1. These terms were used to screen articles for inclusion and to shape the data extraction phase of the review (Table 1).

#### 3.1.3. Context

This review is primarily concerned with information and evidence that is focused on the governance of the disability workforce aligned to allied health in regional, rural, or remote contexts in Australia and in other countries.

Whilst the review is intended to identify successful models of governance of the disability therapy support workforce aligned to allied health in rural and remote settings, the information and evidence base in this context is limited. To ensure all relevant governance information is retrieved and used to inform the review, a tiered approach to information retrieval and filtering was employed. As such this review sampled broadly from contexts such as health (Tier 1) and then filtered the information to disability (Tier 2) and then finally to rural and remote contexts (Tier 3) (Figure 1).

#### 3.1.4. People with Disability

The term disability is used to universally describe the workforce in this sector.

### 3.2. Types of Sources

The review considered peer reviewed evidence including experimental and quasi-experimental study designs, observational studies, qualitative studies, systematic reviews, published reports, and evaluations. Studies published in English since 2000 were included. Table 2 describes the search strategy and inclusion criteria in full.

### 3.3. Method

The scoping review was conducted in accordance with the Joanna Briggs Institute methodology for scoping reviews [12].

#### 3.3.1. Search Strategy

An initial limited search of MEDLINE and CINAHL was undertaken to identify articles on the topic using key words identified from key disability and allied health reports and papers.

The initial search strategy combined all four concepts and obtained 67 records. Three reviewers used a decision tree to filter the 67 results found in the initial MEDLINE search. The reviewers identified one relevant paper from the results. As such, a decision was made to broaden the search to two sets of combined concepts: concept 1 (therapy) support workers (removing the terms “allied health” as this was found to be too restrictive) and concept 3 disability; then, concept 1 (therapy) support workers and concept 4 rural and remote.

The final search strategy (Table 2) located both published and unpublished studies. The text words contained in the titles and abstracts of relevant articles and the index terms used to describe the articles were used to further develop the full search strategy (see Additional File S1). The search strategy, including all identified keywords and index terms, was adapted for each included information source. Due to resource constraints, the reference lists of all studies selected were not screened for additional studies.

#### 3.3.2. Information Sources

Information sources include electronic databases and other electronic search engines. The most recent search was conducted in May 2020.

The databases searched include the following: CINAHL; EMBASE; Web of Science; InfoRMIT: Health Collection; MEDLINE.

#### 3.3.3. Study Selection

**Figure 2:**
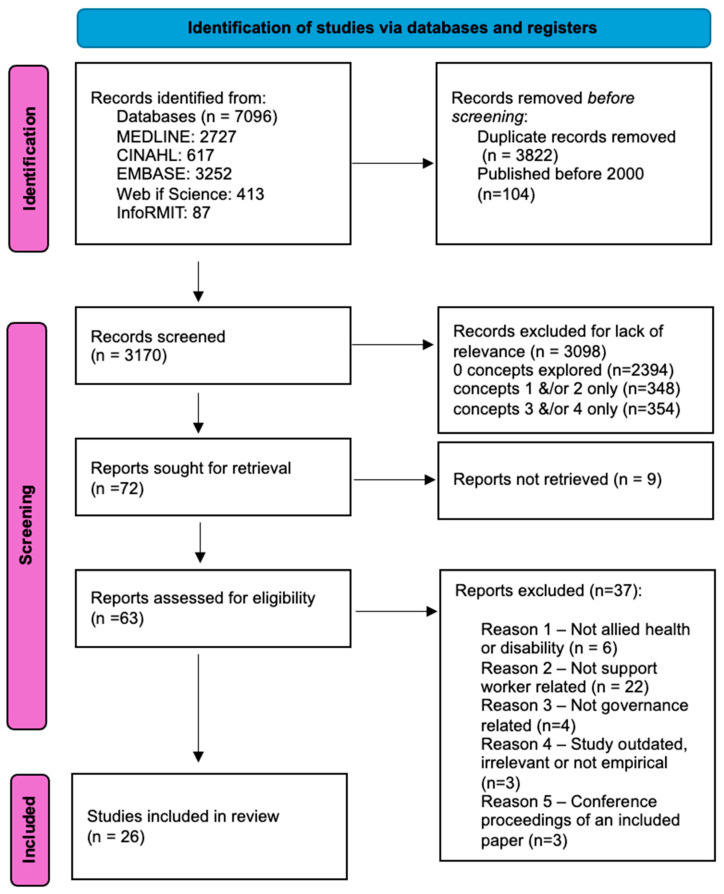
PRISMA-ScR.

Following the search, all identified citations were collated and uploaded into EndNote version 9 and duplicates were removed. To ensure the consistency and accuracy of the screening, a random selection of *n* = 20 titles and abstracts were screened by three reviewers for inclusion using terms outlined in Table 1 and a decision tree (Table 3). When agreement was reached regarding inclusion and exclusion parameters of the decision tree, one reviewer (A.M.) completed screening of all remaining abstracts. Potentially relevant studies were retrieved in full, and their citation details imported into Excel (Microsoft Corporation, Washington, DC, USA). The full text of selected citations was assessed in detail against the inclusion criteria by two reviewers (A.M., G.J.), with the third reviewer (K.B.) confirming any excluded articles. Reasons for exclusion of full text studies that did not meet the inclusion criteria were recorded and are reported in the Preferred Reporting Items for Systematic Reviews and Meta-analyses, Scoping Review (PRISMA-ScR) flow diagram (Figure 2). Any disagreements that arose between the reviewers at each stage of the study selection process were resolved through discussion; there was no need for a fourth reviewer. The results of the search are reported in full in the PRISMA-ScR flow diagram (Figure 2) [25].

#### 3.3.4. Data Extraction

Data were extracted by two reviewers (A.M., G.J.) using a data extraction tool (Additional File S1). The data extracted included specific details about the population, concept, context, study methods, and key findings relevant to the review objective using program logic headings: contexts, interventions, mechanisms, and impact/outcomes [10]. The data extraction tool was constructed by all three reviewers by jointly extracting data from one paper using the above descriptors. It was modified and revised as necessary during the process of extracting data from each included study. The Mixed Methods Appraisal Tool (MMAT) [26] and JBI critical appraisal tools were used to assess the quality of the evidence retrieved. NVivo Version 12 (QSR International, Burlington, MA, USA) was used to extract data and develop a framework for summarizing information. Excel was used to summarize findings from extracted data.

#### 3.3.5. Data Presentation

The extracted data is presented in diagrammatic and tabular form in a manner that aligns with the objective of this scoping review. A narrative summary accompanies the tabulated and charted results and describes how the results relate to the review questions.

## 4. Results

After removal of 3926 duplicates from the original 7096 records, screening of 3170 abstracts led to inclusion of 26 full text papers (see Figure 2, PRISMA). The majority of the papers (*n* = 18) related to all four review concepts, with the remaining eight papers addressing all but the rural concept. Australian papers dominated the evidence base (*n*  =  12), as did qualitative methodologies (*n* = 12) (Table S4, Additional File S2).

In terms of governance interventions, qualitative studies describing support worker capabilities dominated the retrieved evidence, followed by studies describing training, assessment, credentialling, and/or competency requirements around technical, clinical, and administrative skills and knowledge. No papers described a model of governance in full. The quality of papers included in this review ranged from high quality (*n* = 8) to moderate (*n* = 8) to low (*n* = 9). Characteristics of all included studies, including details of the quality assessment, can be found in full in Additional File S2.

### 4.1. What Are the Key Components of Governance Models/Interventions for Disability Therapy Support Workers in Rural and Remote Settings?

Soft skills and knowledge were the most common governance elements reported in the retrieved evidence (*n* = 17 papers). Specific skills and knowledge (competencies), assessment of competencies, credentialling, supervision and support of support workers, use of care plans, and continuing professional development were also described (Table 4).

#### 4.1.1. Capabilities

The term capability was used to summarize qualitative descriptions relating to soft skills and knowledge of disability therapy support workers. Capabilities are defined as “non-clinical skills, knowledge and attributes that show work is being done well… Capability development involves building knowledge, skills and attributes to complement the clinical and technical skills allied health professionals already have. Capabilities help practitioners to provide high quality services and to navigate the services system” [41] (p. 12).

Capabilities identified in the evidence fell into two categories: soft skills and knowledge and intrinsic attributes. Soft skills and knowledge dominated the retrieved evidence (Table S6, Additional File S2. These included (in order of frequency of citation) decision making skills, skills around building relationships with clients, advocating for clients, communication and interaction skills, risk assessment and risk management skills, skills around building relationships with multiple stakeholders (including health professionals), adapting to client needs/client-centred care, counselling, critical thinking/clinical judgement/clinical reasoning, providing clients with emotional support, negotiating role boundaries, empowering clients, and providing personal and compassionate support.

Notably, decision making and assessing and managing risk were identified as soft skills and were not necessarily found within articles that examined training of more technical skills and knowledge (competencies). Several papers identified the complex interplay between capabilities around decision making and managing risk and the need for other capabilities such as clinical reasoning and critical judgement [42,47], advocacy [27], adapting to client needs [29,42], and awareness of self in supporting decision making (epistemic authority) [27,32]. Another paper describes the complex interplay between the essential role of advocacy and the need for appropriate decision-making capabilities for clients [29].

Intrinsic attributes, which included having a caring nature and being patient, honest, and empathetic, having personal integrity, compassion, a sense of equity and social justice, as well as a passion for the work, were less frequently cited in the evidence [27,36,42].

#### 4.1.2. Competence

A small number of papers (*n* = 8) described aspects of technical skills and knowledge within the disability support workforce (Table S7 and S7A, Additional File S2). These were grouped under the term competency.

‘Competency is the consistent application of knowledge and skill to the standard of performance required in the workplace. It embodies the ability to transfer and apply skills and knowledge to new situations and environments’ [52]. For the purpose of this review, the term competence refers to the knowledge and skills needed to support clinical competency for specific work roles or practices of the disability therapy support workforce. Information extracted from included papers related to four groups of competencies: therapy-focused, such as home visit skills and knowledge; administrative- and management-focused, such as skills around referring to other agencies or disciplines; disability-focused, such as personal care skills and knowledge; more specialist skills and knowledge, such as management of dysphagia. The weight of evidence, though small, was around the administrative- and management-focused competencies, which included (in order of most frequently cited) technical competence in referring to other therapies/services/agencies, record keeping, organizational and business management skills and knowledge, legal and ethical standards, and customers service skills and knowledge.

#### 4.1.3. Supervision, Support, Assessment of Competency, Credentialling and Care Plans

The remainder of the retrieved evidence described aspects of governance interventions such as credentialling (*n* = 4 papers), supervision (*n* = 3), formal assessment of competence (*n* = 2), support structures (*n* = 2), the use of care plans (*n* = 1), and professional development (*n* = 1).

### 4.2. What Contexts and Mechanisms Are Required to Successfully Apply Governance Models for Disability Support Workers in Rural and Remote Settings?

#### Contexts

Data describing enabling and disabling contexts for governance components were identified across 18 of the retrieved papers. These included the following (in order of most to least frequently cited) (Table S8, Additional File S2): consistent and sufficient staffing; clear job and role descriptions; whole-of-community approach/multisector coordination; training and professional development is routinely provided, resourced, and supported; support worker knowledge and skills are valued; staff are provided the time, resources, and opportunity to look after their wellbeing; sufficient time is available to staff to develop relationships/negotiate role boundaries; clear lines of communication and accountability; culture of organization is relaxed/playful and supportive; complex funding models [disabling context]; encouraging and supportive leadership style; safety precautions incorporated into training protocols; enablement culture; AHPs involved in developing training packages; project resourcing; clear accountability and liability around training assessment; acknowledging complexity of the supervision/workplace context (rural); NDIS—patient-centred focus; size of organization.

Several authors describe the need for consistent and sufficient staffing as an enabling context for governance components to be successful. Ennion et al. [36], for example, describe how the combination of several (disabling) contexts leads to specific difficulties in providing access to quality rehabilitation services. Specifically, these disabling contexts included poorly defined roles, lack of resourced training, a high turnover of support workers, and poor staff retention. Forster, S. and Iacono, T. [37] reiterate the importance of providing sufficient time [enabling context] for DTSW to develop complex and meaningful communication styles and therefore build relationships with [governance components] an intellectually disabled person. They also describe the importance of organizations valuing [enabling context] the soft skills and personal attributes [governance components] that are already held by DTSW prior to any training being undertaken. Papers examining low-resource settings also frequently described the need for multi-sectorial or whole-of-community support to ensure that any governance intervention (training model, supervision, new worker) is successful and sustainable [35,38,39,48].

Moskos and Isherwood [47] describe how the NDIS, as an enabling context, has led to a shift in the governance components required of support staff. With a focus on individualized funding and person-centred care, respondents in their study perceived that there was a shift away from task-oriented skills and competencies towards more client-focused, goal-orientated skills and competencies “… This means workers now must look at what a customer wants to do and be responsive to their wishes rather than working from a service directed plan” [47] (p. 44).

### 4.3. Mechanisms

Mechanisms refers to the enabling or disabling activities for the identified governance components to be successful (Table S9, Additional File S2). The mechanisms extracted from the retrieved evidence fell into the same categories as the key governance components identified: capability mechanisms; training mechanisms; competency mechanisms; supervision, assessment, and credentialling mechanisms.

#### 4.3.1. Capability Mechanisms

Capability mechanisms are characterized by a multitude of soft skills that, when demonstrated by the support worker, allow for governance principles to be realized (Table S9, Additional File S2). It is the capable undertaking of these soft skills that enables the realization of goals for people with disabilities. This was demonstrated by Chappel and Johannsmeier [35] in their study examining the impact of community-based rehabilitation in South Africa. The authors describe how the support worker advisor and counselling skills, alongside relationship-building skills, led to improved self-esteem, self-confidence, and that the good relationships developed between support workers and persons with disability (PWD) appeared to give PWD the necessary motivation and encouragement to achieve their own personal goals.

Bourke et al. [32] similarly discuss the need for DTSW and people with spinal cord injury who are newly returned home from hospital to negotiate their role boundaries through establishing ground rules and negotiating relationships. In this paper, the enabling mechanisms of relationship building and negotiating role boundary capabilities are necessary in order to “develop familiarity and boundaries” in an uncertain experience for the client [32] (p. 260).

#### 4.3.2. Successful Capability Is Facilitated by the following Mechanisms

Self-reflection/awareness of self in decision-making process with a person with disability; recognizing inherent attributes may be more important in some instances than trained capabilities; client relationship building (trust); negotiating boundaries; developing counselling/advisory roles; multifaceted relationship building; professional relationship building; complex communication skills; advocacy and empowerment skills; critical thinking and clinical judgement; emotional support; compassionate support; decision making skills; critical thinking skills; communication skills; skills around administration of paperwork; personal attributes and behaviours; client-focused care; risk management; flexibility.

Successful training is facilitated by mechanisms such as the following: a needs assessment prior to training being undertaken so that training could be tailored to participants; the use of technology such as smart phones, video recordings/video modules, videoconferencing; the combination of practical and theory sessions; opportunity for clarification and networking; individualized training using modelling and personalized written guidelines; practical observation and application of learned techniques/theory; immersion into the working environment with support available at regular intervals (train-work-train model); utilizing a train the trainer model.

One study of a training program for therapy assistants in rural Australia describes how a needs assessment survey was distributed to therapy assistants and supervising allied health professionals to identify training priorities. This assessment confirmed both a widespread need for training and specifically that clinical training in the area of paediatrics was identified as a priority [50]. The same study also identifies the combination of enabling mechanisms technology (video modules) to deliver training and a train the trainer approach. The advantage of using video technology was that each training package contained all the resources necessary for any allied health professional to present the same module in a different setting to a different audience of therapy assistants.

Hoyle et al.’s [41] literature review of education interventions for clinically invasive interventions reiterates that while the methods for training support workers are important, so too are capabilities around critical thinking “competence should include not only the skills and knowledge but also critical thinking, clinical judgment, the formulation of attitudes, and the determination of the feasibility of an action” [41] (p. 246).

#### 4.3.3. Successful Governance Is Facilitated by Competency Mechanisms

These mechanisms are characterized by enabling technical tasks or activities carried out by support workers. These include the following: skills and knowledge to carry out multiple practical and technical skills; multidisciplinary interventions; skills and knowledge to maintain hygiene standards; skills and knowledge to maintain legal and ethical standards; skills and knowledge to carry out an enabling philosophy of care; skills and knowledge to contribute to a disability organization’s success; skills and knowledge to deliver customer-service and person-centred care; skills and knowledge to facilitate access to other services or to refer to other agencies or health professionals.

Wark et al. [51] describe how the mechanism of competence in facilitating access to appropriate aged care services for individuals ageing with learning disabilities is so important to client outcomes given the significant complexity of the aged and disability funding sectors and significant lack of appropriate aged care services in the rural context for individuals ageing with a learning disability: “It has been recognized that the planning of services for this group needs to be proactive and designed to deliberately facilitate access … However, determining what services are appropriate, and then how to access them in Australia, is confused by the number of differing departments within the different levels of government that oversee service delivery for individuals ageing with learning disability”. Again, Wark et al. [51] describe how competency mechanisms interplay with capability mechanisms (advocacy) to achieve outcomes for individuals ageing with a learning disability in a rural setting: “Many of the issues raised by the participants related to accessing generic community-based services or specialist medical support. Exemplar items for this theme include advocating for clients to get appropriate medical care; gaining access to appropriate recreational and leisure activities in retirement such as day services programs; accessing appropriate professional support; and advocating for clients to get appropriate aged-care services” [51] (pp. 217–218).

#### 4.3.4. Successful Supervision Is Facilitated by the following Mechanisms

The use of discussion on client progress; observation of practice by the supervisor face to face or via video recording; group supervision; 1:1 supervision; demonstration of a new program or when significant changes are made to an existing program. Importantly supervision needs to be set out in clear, systematic guidelines which specify the frequency, timetabling requirements, and methods for supervision (face to face, telephone, videoconferencing, video recording) and feedback. As described by Lin and Goodale [45], it is also imperative to monitor and record (log) the frequency and quality of supervision. The supervisor needs to have received training and to have been involved in devising the supervision strategy or training intervention(s).

#### 4.3.5. Successful Formal Assessment of Competence and Credentialling Are Facilitated by the following Mechanisms

Partnerships with recognized, formalized training organizations; external certification that is recognized by multiple agencies; undertaking of baseline and follow-up knowledge assessments; evaluation of the impact of education on participants; legal accountability for competency attainment.

Hoyle et al. [41] describe, in their review of education interventions for clinically invasive interventions, that there was no consistent method for evaluation and none of the studies evaluated the long-term impact of education or the impact it has had on the service users themselves. Furthermore, none of the studies addressed responsibility or accountability when support workers undertake the tasks once they had been educated. They suggested that consideration needs to be given to the issue of liability if the delivery of a training session goes wrong and that this needs to be addressed prior to the commencement of an educational program.

### 4.4. Success and Impact

Measures of success and impact were sparsely described in the evidence (Tables S4 and S9, Additional File S2). Where a particular governance intervention or element was described (or the lack thereof), outcomes were broadly described in terms of the impact on capacity to meet client needs and outcomes (*n* = 9); support worker and professional staff outcomes (*n* = 27); and organizational or community outcomes (*n* = 5).

Most evidence described the impact of a governance intervention in terms of improved staff skills, knowledge, or capability (*n* = 12) such as improved technical knowledge, improved advocacy skills, etc.; improved (or reduced) (*n* = 8) staff wellbeing such as feeling valued and belonging or conversely feeling emotional conflict and stress; and improved (or reduced) relationships between staff (*n* = 6). Where client outcomes were described, governance interventions (or lack of) led to improved (or reduced) engagement with services, inclusion in activities/therapy, and continuation of/disruption to service provision.

### 4.5. Strength and Quality of the Evidence

The quality of included evidence was rated as moderate to good overall, with qualitative studies displaying the highest quality ratings and quantitative studies displaying the lowest quality ratings (Table S4, Additional File S2). There was a significant lack of outcome level data and due to the heterogeneity of methods and interventions studies, there was also significant inconsistency in the reporting of impact and outcomes. As such, the capacity to link governance contexts and mechanisms with outcomes to devise a ‘success’ logic framework was compromised, and a final logic model was unable to be completed with a level of certainty.

## 5. Discussion

This scoping review has systematically examined a broad range of peer reviewed evidence to identify and understand the contexts and mechanisms that lead to successful governance of disability support/therapy workers who deliver allied health interventions in rural and remote areas.

In summary, this review has identified that a successful governance disability support/therapy worker model for delivering allied health interventions is defined as one that optimally supports the capability of its support (and professional) workforce. Capability is characterized by a multitude of soft skills and knowledge and intrinsic attributes that, when demonstrated by the support worker, allow for governance principles to be realized. It is the capable undertaking of soft skills and knowledge combined with particular attributes that enables DTSW to support the realization of goals for people with disabilities. Capabilities were described as follows (in order of frequency of citation):

- Decision making skills;
- Building relationships with clients;
- Advocating for clients;
- Communication and interaction skills;
- Risk assessment and risk management skills;
- Building relationships with multiple stakeholders;
- Adapting to client needs/client-centred care;
- Counselling;
- Critical thinking/clinical judgement/clinical reasoning;
- Providing clients with emotional support;
- Negotiating role boundaries;
- Empowering clients;
- Providing personal and compassionate support.

The evidence suggests that to optimize the capability of the support workforce, a disability support/therapy worker governance model should include attention to the following 18 parameters (presented in order of most to least reported):

- Consistent and sufficient staffing;
- Clear job and role descriptions for all staff;
- Whole of community approach/multisector coordination to delivery of care;
- Training and professional development is routinely provided, resourced and supported;
- Support worker knowledge and skills are valued;
- Staff are provided time, resources, and opportunity to look after their wellbeing;
- Sufficient time is made available to staff to develop relationships/negotiate role boundaries;
- Clear lines of communication and accountability;
- Culture of organization is relaxed/playful and supportive;
- Simplified funding models;
- Encouraging and supportive leadership style;
- Safety precautions incorporated into training protocols;
- Enablement culture;
- AHPs involved in developing training packages;
- Project resourcing;
- Clear accountability and liability around training assessment;
- Acknowledging complexity of the supervision/workplace context (rural).

To realize success, a suite of mechanisms is described which include evidence-based strategies that a rural disability organization should consider employing when constructing their governance model to optimize the conditions for success.

These include attention to capability mechanisms (*n* = 20) such as tools for self-reflection/awareness of self in decision-making processes with a person with disability and negotiating boundaries; training mechanisms (*n* = 8) such as ensuring a needs assessment occurs prior to training being undertaken so that training can be tailored to participants and implementing train the trainer models; competence mechanisms (*n* = 8) such as structured ways to enhance skills and knowledge to carry out multiple practical and technical skills and multidisciplinary interventions; supervision mechanisms (*n* = 8) such as the use of discussion on client progress and observation of practice by the supervisor face to face or via video recording; formal assessment of competence and credentialling mechanisms (*n* = 5) such as partnerships with recognized, formalized training organizations and external certification that is recognized by multiple agencies.

### Limitations

Despite a broad search of the evidence base, there is extremely limited literature that has specifically examined all four concepts under review: governance models for therapy support workers who work with people with disabilities in rural contexts. This is not surprising and was identified as a risk from the outset, hence the broad search criteria and inclusive search strategy and scoping review methodology. However, of concern and for future research attention is the fact that the two articles that were identified in the review process that specifically related to therapy support workers in rural areas [50,52] were assessed as low-quality research by the research team as they were evaluations only. Unfortunately, this is not an uncommon situation given the very low resourcing and investment in rural research, in particular in applied and allied health research [53,54].

Future rural research needs to be funded and needs to focus on how best to ensure that these small but important micro-professional groups [55] are provided with the best contexts and opportunities to effectively and safely improve the wellbeing and lives of rural Australians who live with a disability.

Governance is commonly used as a risk management tool; however, none of the papers included in this review explicitly explored risk management as a concept or reported outcomes specifically relating to risk. Instead, a number of papers described risk management as either an inherent quality that is contained within a support worker’s capability profile (reported as relating to decision making skills or capability of making a risk assessment [29,46,47,49]) or relating to a set of capabilities such as specific clinical or technical skills [41] or knowledge of best practice and ethics [47] (Table S7A, Additional File S2). Given the challenges governing a workforce that is so widely dispersed in rural regions, research that specifically examines the relationship between DTSW risk-associated capabilities and the outcomes of care for PWD is an area where future research should focus.

Finally, only seven of the included papers sought user perspectives on factors relating to governance of the DTSW workforce [28,32,35,36,44,46,47]. Given the focus of the NDIS on user engagement and user-driven care, the evidence base would benefit from research that specifically seeks user perspectives of what comprises good governance and how this might impact on outcomes of importance to them.

## 6. Conclusions

Effective governance models for DTSW in rural and remote settings require a combination of soft skills, technical competencies, supportive contexts, and structured mechanisms. Enhancing soft skills (capabilities), training, and supervision while considering contextual factors can lead to improved staff performance, client outcomes, and organizational effectiveness. Further research is needed to evaluate the long-term impact of governance interventions and ensure accountability in education programs.

## Figures and Tables

**Table 1:** Search and screening terms for each concept.

Concept 1: Therapy Support Workers Who Deliver Allied Health Support	Concept 2: Governance	Concept 3: Disability	Concept 4: Rural and Remote
Therapy support workers who deliver allied health support	Governance	Disability	Rural and Remote
Assistant practitioner *.mp or	Governance	Disability * therapy.mp or	Rural health/or
Community health workers/or	Quality	Disabled persons/or	Rural health services.mp or
Auxiliary personnel *.mp or	Safe (ty)	Disabled people.mp or	Rural population.mp or
MM Allied health personnel or	Effective	Disabled persons/or	MM Rural Health Personnel or
Allied health workers.mp or	Risk	Disability * service *.mp or	MM Rural Health Centers OR
Allied health assistant *.mp or	Accountable/accountability	MM “Health Services for Persons with Disabilities”	MM “Services for Australian Rural and Remote Allied Health or
Allied health helper *.mp or	Person-centred		Rural
Allied health aide *.mp or	Complaint		
Personal care workers.mp or	Incident		
Personal care assistant *.mp or	Misconduct		
Patient care workers.mp or	Neglect		
Personal care helper *.mp or	Violence		
Personal care aide *.mp or	Abuse		
Patient care helper *.mp or	Supervision		
Patient care aide * or	Delegation		
Patient care assistant *.mp or	Allocating tasks		
Support worker *.mp or	Training		
Support helper *.mp or	Competent/competency		
Support aide *.mp	Transferring tasks		
Health worker *.tw or	Qualification		
Health assistant *.tw or	Responsibility/responsibilities		
Health worker *.mp or	Knowledge		
Health assistant *.mp or	Skills		
Community rehabilitation helper *.mp or	Development		
Community rehabilitation worker *.mp or	Professional		
Community rehabilitation assistant *.mp or	Development		
Community rehabilitation aide *.mp or	Skill level		
Community health helper *.mp or	Credential		
Community health worker *.mp	Communication		
Community health assistant *.mp or	Education		
Community health aide *.mp or	Instruction		
Therapy support worker *.mp or	Assess		
Disability * support worker *.mp or			
Workforce/or			
Health Workforce.mp			
MM “Physical Therapist Assistants” or			

* Terms used during abstract screening (taken from Additional File S1).

**Table 2:** STARLITE † search strategy.

Process	Detail
Sampling strategy	Selective: samples databases from medicine, nursing, allied health, and social science fields within specified limits.
Type of study	All, quantitative research (randomized controlled trial, controlled clinical trial, controlled before and after study, uncontrolled before and after study), qualitative research (grounded theory, ethnography, action research, exploratory approaches, phenomenology, and systematic reviews). Published reports, evaluations, guidelines, and frameworks.
Approaches	Subject searching, citation searching, contact with authors.
Range of years	January 2000–May 2020.
Inclusion and exclusions ¶	Inclusion: empirical study or published report, evaluation, framework, or guideline describing governance models pertaining to allied health assistants, therapy assistants, and disability therapy support workers who deliver care and interventions under the guidance of allied health professionals and specifically in disability contexts (Tier 2) and in regional, remote, and/or rural areas (Tier 3).
Terms used	See concept map (Table 1)
Electronic sources	CINAHL; EMBASE; InfoRMIT:Health Collection; MEDLINE and Web of Science.

† Adapted from STARLITE principles for reporting systematic literature reviews [24]; ¶ detailed in Table 3 decision tree.

**Table 3:** Decision tree/evidence screening process.

Process	Decision		
1. Does the paper examine the assistant/support workforce (Concept 1)?	Yes—Go to 2	No—Exclude	Cannot tell—Get full paper
2. Does the paper relate to the allied health professions (outside of acute health or rehabilitation settings) (Concept 1)?	Yes—Go to 3	No—Exclude	Cannot tell—get full paper
3. Does the paper examine a governance model, framework, policy, intervention, or evaluation (Concept 2)?	Yes—Go to 4	No—Exclude or consider for background	Cannot tell—Get full paper
4. Does the study provide detail of the governance model or intervention (Concept 2)?	Yes –Include as Tier 1 evidence and go to 5	No—Exclude or consider for background	Cannot tell—Get full paper
5. Does the paper relate to the disability sector (Concept 3)?	Yes—Include as Tier 2 evidence and go to 6	No—Include paper for Tier 1 evidence	Cannot tell—Get full paper
6. Does the study examine regional, rural and/or remote areas (Concept 4)?	Yes—Include as Tier 3 evidence	No—Include paper for Tier 1 or 2 evidence and check references	Cannot tell—Get full paper

**Table 4:** Governance components described in retrieved evidence.

Reference	Governance Component								
	Capability (Soft Skills)	Capability (Intrinsic Attributes)	Competency	Supervision	Credentialling	Assessment of Competence	Professional Development	Support Structures	Care Plans
[27]	X								
[28]	X			X					
[29]	X	X							
[30]									
[31]					X				
[32]	X	X							
[33]	X								
[34]								X	
[35]	X								
[36]	X		X						
[37]	X								
[38]	X	X	X	X				X	
[39]			X		X				
[40]									
[41]	X		X		X	X	X		X
[42]	X								
[43]	X								
[44]	X		X						
[45]				X					
[46]	X								
[47]	X	X	X						
[48]						X			
[49]	X								
[50]			X		X				
[51]	X		X						

X indicates the governance component.

## Data Availability

The original contributions presented in the study are included in the article/Supplementary Materials, further inquiries can be directed to the corresponding author/s.

## Supplementary Materials

The following supporting information can be downloaded at: https://www.mdpi.com/article/10.3390/ijerph21060693/s1, Additional File S1, Additional File S2.

## References

[1] Disability Reform Council (2005). National Disability Insurance Scheme Integrated Market, Sector and Workforce Strategy.

[2] National Disability Services (2018). State of the Disability Sector Report.

[3] Mucic A. (2019). Building a Workforce Using Allied Health Assistants. https://www.disabilityservicesconsulting.com.au/resources/building-workforce-allied-health-assistants.

[4] NSW Ministry of Health (2013). Allied Health Assistant Framework. NSW Government. http://www1.health.nsw.gov.au/pds/ActivePDSDocuments/GL2013_005.pdf.

[5] Department of Health and Human Services (2018). Supervision and Delegation Framework for Allied Health Assistants and the Support Workforce in Disability. https://www2.health.vic.gov.au/-/media/health/files/collections/policies-and-guidelines/a/allied-health-in-disability---supervision-and-delegation-framework.pdf?la=en&hash=17C8B0DA1306D5944508699B58349590B1BE4CCA.

[6] Main R. (2018). Implementation of the National Disability Insurance Scheme and the Provision of Disability Services in New South Wales.

[7] World Health Organisation (2001). International Classification of Functioning, Disability and Health.

[8] Moran A., Enderby P., Nancarrow S. (2011). Defining and identifying common elements of and contextual influences on the roles of support workers in health and social care: A thematic analysis of the literature. J. Eval. Clin. Pract..

[9] Braithwaite J., Travaglia J.F. (2008). An overview of clinical governance policies, practices and initiatives. Aust. Health Rev..

[10] Pawson R., Tilley N. (1997). Realistic Evaluation.

[11] Baxter S., Killoran A., Kelly M., Goyder E. (2010). Synthesizing diverse evidence: The use of primary qualitative data analysis methods and logic models in public health reviews. Public Health.

[12] Peters M., Godfrey C., McInerney P., Baldini S., Khalil H., Parker D., Aromataris E., Munn Z. (2017). Chapter 11: Scoping Reviews. Joanna Briggs Institute Reviewer’s Manual.

[13] Allied Health Professions Australia [AHPA] (2019). What Is Allied Health?. https://ahpa.com.au/what-is-allied-health/.

[14] Sevices for Australian Rural and Remote Allied Health (SARRAH) (2007). A Framework for the Classification of the Health Professional Workforce.

[15] Greater Northern Australia Regional Training Network (2013). Project Report: Rural and Remote Generalist—Allied Health Project.

[16] Keane S., Smith T.N., Lincoln M., Wagner S.R., Lowe S.E. (2008). The rural allied health workforce study (RAHWS): Background, rationale and questionnaire development. Rural Remote Health.

[17] Nancarrow S.A., Roots A., Grace S., Moran A.M., Vanniekerk-Lyons K. (2013). Implementing large-scale workforce change: Learning from 55 pilot sites of allied health workforce redesign in Queensland, Australia. Hum. Resour. Health.

[18] Services for Australian Rural and Remote Allied Health (SARRAH) (2019). The Allied Health Rural Generalist Pathway.

[19] Services for Australian Rural and Remote Allied Health (SARRAH) (2016). Position Statement: Allied Health Professions and Rural Generalism.

[20] Retief M., Letsosa R. (2018). Models of disability: A brief overview. Hts Teol. Stud. Theol. Stud..

[21] Scally G., Donaldson L.J. (1998). The NHS’s 50 anniversary. Clinical governance and the drive for quality improvement in the new NHS in England. BMJ.

[22] (2018). National Disability Insurance Scheme (Quality Indicators) Guidelines 2018.

[23] National Disability Insurance Scheme Quality and Safeguards Commission (2018). NDIS Code of Conduct (NDIS Providers).

[24] Booth A. (2006). “Brimful of starlite”: Toward standards for reporting literature searches. J. Med. Libr. Assoc..

[25] Moher D., Liberati A., Tetzlaff J., Altman D.G., PRISMA Group (2009). Preferred Reporting Items for Systematic Reviews and Meta-Analyses: The PRISMA Statement. PLoS Med..

[26] Hong Q.N., Pluye P., Fabregues S., Bartlett G., Boardman F., Cargo M., Dagenais P., Gagnon M.-P., Griffiths F., Nicolau B. (2018). Mixed Methods Appraisal Tool (MMAT) Version 18: User Guide.

[27] Antaki C., Webb J. (2019). When the larger objective matters more: Support workers’ epistemic and deontic authority over adult service-users. Sociol. Health Illn..

[28] Asher L., Hanlon C., Birhane R., Habtamu A., Eaton J., Weiss H.A., Patel V., Fekadu A., De Silva M. (2018). Community-based rehabilitation intervention for people with schizophrenia in Ethiopia (RISE): A 12 month mixed methods pilot study. BMC Psychiatry.

[29] Banks S. (2016). “Knowing me, knowing you’: Disability support worker as emotional mediator?. Sexualities.

[30] Bhattacharjya S., Lenker J. (2019). Using Smartphones to Disseminate Video-Based Rehabilitation Training Materials in Resource-Poor Regions in India. Arch. Phys. Med. Rehabilitation..

[31] Bouchonville M.F., Hager B.W., Kirk J.B., Qualls C.R., Arora S. (2018). Endo echo improves primary care provider and community health worker self-efficacy in complex diabetes management in medically underserved communities. Endocr. Pract..

[32] Bourke J.A., Nunnerley J.L., Sullivan M., Derrett S. (2019). Relationships and the transition from spinal units to community for people with a first spinal cord injury: A New Zealand qualitative study. Disabil. Health J..

[33] Bourne J., Selman M., Hackett S. (2020). Learning from support workers: Can a dramatherapy group offer a community provision to support changes in care for people with learning disabilities and mental health difficulties?. Br. J. Learn. Disabil..

[34] Brooker J., Julian J., Webber L., Chan J., Shawyer F., Meadows G. (2013). Evaluation of an Occupational Mindfulness Program for Staff Employed in the Disability Sector in Australia. Mindfulness.

[35] Chappell P., Johannsmeier C. (2009). The impact of community based rehabilitation as implemented by community rehabilitation facilitators on people with disabilities, their families and communities within South Africa. Disabil. Rehabil..

[36] Ennion L., Rhoda A. (2016). Roles and challenges of the multidisciplinary team involved in prosthetic rehabilitation, in a rural district in South Africa. J. Multidiscip. Healthc..

[37] Forster S., Iacono T. (2008). Disability support workers’ experience of interaction with a person with profound intellectual disability. J. Intellect. Dev. Disabil..

[38] Gilmore B., MacLachlan M., McVeigh J., McClean C., Carr S., Duttine A., Mannan H., McAuliffe E., Mji G., Eide A.H. (2017). A study of human resource competencies required to implement community rehabilitation in less resourced settings. Hum. Resour. Health.

[39] Goodale B., Spitz S., Beattie N., Lin I. (2007). Training rural and remote therapy assistants in Western Australia. Rural Remote Health.

[40] Haines D., Wright J., Comerasamy H. (2018). Occupational Therapy Empowering Support Workers to Change How They Support People with Profound Intellectual and Multiple Disabilities to Engage in Activity. J. Policy Pract. Intellect. Disabil..

[41] Hoyle L., Brown M., Donaldson J., Karatzias T. (2017). Invasive Clinical Intervention Education for Social Care Support Workers of Adults: A Review of the Current Literature. J. Policy Pract. Intellect. Disabil..

[42] Hussain R., Wark S., Muller A., Ryan P., Parmenter T. (2019). Personal relationships during end-of-life care: Support staff views of issues for individuals with intellectual disability. Res. Dev. Disabil..

[43] Iacono T., Davis R., Humphreys J., Chandler N. (2003). GP and support people’s concerns and priorities for meeting the health care needs of individuals with developmental disabilities: A metropolitan and non-metropolitan comparison. J. Intellect. Dev. Disabil..

[44] Iacono T., Humphreys J., Davis R., Chandler N. (2004). Health care service provision for country people with developmental disability: An Australian perspective. Res. Dev. Disabil..

[45] Lin I., Goodale B. (2006). Improving the supervision of therapy assistants in Western Australia: The Therapy Assistant Project (TAP). Rural Remote Health.

[46] Mason V., Williams V. (2017). Enabling good emotional support for and with people with learning disabilities. Tizard Learn. Disabil. Rev..

[47] Moskos M., Isherwood L. (2019). Individualised funding and its implications for the skills and competencies required by disability support workers in Australia. Labour Ind. A J. Soc. Econ. Relat. Work..

[48] Narayan J., Pratapkumar R., Reddy S.P. (2017). Community managed services for persons with intellectual disability: Andhra Pradesh experience. J. Intellect. Disabil..

[49] Venema E., Vlaskamp C., Otten S. (2018). The role of volunteers in the social integration of people with intellectual disabilities. Res. Pract. Intellect. Dev. Disabil..

[50] Wark S., Hussain R., Edwards H. (2013). Rural and Remote Area Service Provision for People Aging With Intellectual Disability. J. Policy Pract. Intellect. Disabil..

[51] Wark S., Hussain R., Edwards H. (2015). Assisting individuals ageing with learning disability: Support worker perspectives. Tizard Learn. Disabil. Rev..

[52] Australian Commission on Safety and Quality in Health Care (2014). National Safety and Quality Health Service Standards: Training and Competencies for Recognising and Responding to Clinical Deterioration in Acute Care.

[53] O’Sullivan B.G., Cairns A., Gurney T.M. (2020). Understanding the field of rural health academic research: A national qualitative, interview-based study. Rural Remote Health.

[54] Moran A., Haines H., Raschke N., Schmidt D., Koschel A., Stephens A., Opie C., Nancarrow S. (2019). Mind the gap: Is it time to invest in embedded researchers in regional, rural and remote health services to address health outcome discrepancies for those living in rural, remote and regional areas?. Aust. J. Prim. Health.

[55] Nancarrow S.A., Borthwick A.M. (2021). The Allied Health Professions: A Sociological Perspective.

